# Signals Can Trump Rewards in Attracting Seed-Dispersing Ants

**DOI:** 10.1371/journal.pone.0071871

**Published:** 2013-08-13

**Authors:** Kyle M. Turner, Megan E. Frederickson

**Affiliations:** Department of Ecology & Evolutionary Biology, University of Toronto, Toronto, Ontario, Canada; Centro de Investigación y de Estudios Avanzados, Mexico

## Abstract

Both rewards and signals are important in mutualisms. In myrmecochory, or seed dispersal by ants, the benefits to plants are relatively well studied, but less is known about why ants pick up and move seeds. We examined seed dispersal by the ant *Aphaenogaster rudis* of four co-occurring species of plants, and tested whether morphology, chemical signaling, or the nutritional quality of fatty seed appendages called elaiosomes influenced dispersal rates. In removal trials, ants quickly collected diaspores (seeds plus elaiosomes) of *Asarum canadense, Trillium grandiflorum,* and *Sanguinaria canadensis*, but largely neglected those of *T. erectum*. This discrepancy was not explained by differences in the bulk cost-benefit ratio, as assessed by the ratio of seed to elaiosome mass. We also provisioned colonies with diaspores from one of these four plant species or no diaspores as a control. Colonies performed best when fed *S. canadensis* diaspores, worst when fed *T. grandiflorum*, and intermediately when fed *A. canadense, T. erectum,* or no diaspores. Thus, the nutritional rewards in elaiosomes affected colony performance, but did not completely predict seed removal. Instead, high levels of oleic acid in *T. grandiflorum* elaiosomes may explain why ants disperse these diaspores even though they reduce ant colony performance. We show for the first time that different elaiosome-bearing plants provide rewards of different quality to ant colonies, but also that ants appear unable to accurately assess reward quality when encountering seeds. Instead, we suggest that signals can trump rewards as attractants of ants to seeds.

## Introduction

In mutualisms, individuals of different species exchange goods or services to their mutual benefit [1], [2]. Many mutualisms involve multiple species on one or both sides of the interaction [3], so organisms often rely on both signals and direct assessments of rewards to choose whether to interact with a variety of potential partners. In pollination mutualisms, for example, flower-visiting insects may directly evaluate nectar or pollen rewards [4] or they may base their foraging decisions on signals. Signals may be directly associated with reward level (e.g., display size or morphology [5]–[7]) or they may attract pollinators that have learned to associate signals with rewards (e.g., color, odor [8], [9]). But signals open the door to deception: if rewards are costlier than signals to produce, plants may benefit from reducing rewards and instead either misinforming partners of their quality [7], [10] or using signals that trigger out-of-context behaviors, a phenomenon known as “sensory trapping” [11], [12]. These two types of false signals, exemplified in pollination by food deceptive and sexually deceptive orchids, respectively [8], [10], [11], can prompt animals to interact with unrewarding partners.

Myrmecochory, or seed dispersal by ants, is another common example of a multi-specific insect-plant interaction. This partnership is signified in the plant partner (the myrmecochore) by the presence of a lipid-rich food body on the seed known as an elaiosome. Elaiosomes occur in over 11,000 plant species, distributed across 55 angiosperm families, and representing over 100 independent evolutionary origins [13], with the elaiosome derived from a variety of seed structures [14]. Foraging ant workers pick up diaspores (seeds with attached elaiosomes) and carry them back to their nests. Ants remove complete diaspores much more rapidly than they do seeds stripped of elaiosomes [15]–[17], often using the elaiosome as a handle [18]. Once in the nest, ants generally remove and feed elaiosomes to larvae before depositing seeds in a midden pile inside or outside their nest [19], [20]. Myrmecochory may occur as the sole dispersal mode, or following avian or ballistic dispersal (e.g., [21]).

Several studies have measured the benefits of myrmecochory to plants. These benefits may include: dispersal away from the parent plant and reduction of parent-offspring or sibling competition [22]; directed dispersal to favorable microsites such as ant nests or middens [23]; or avoidance of seed predators [24]. Proximately, removal of the elaiosome by ants may trigger germination [25]. The extent to which different ant species provide these benefits can depend on worker size, foraging behaviors, and colony organization; Giladi’s [26] review suggested that several plant traits, including elaiosome size and chemistry, as well as the timing, spatial pattern, and volume of seed release, may be adaptations that allow them to preferentially associate with high-quality dispersers. However, the converse–whether ants make adaptive choices about whether to disperse seeds–is completely unexplored. In fact, very few studies have examined whether ants benefit from myrmecochory.

Many elaiosomes do provide nutrients, such as sterols and linolenic acid, which ants cannot independently synthesize [27]. Elaiosomes are also highly enriched in both amino acids [28] and caloric value [14] compared to the seeds to which they are attached, suggesting that elaiosomes are a high-quality food. However, the few studies examining the effects of consuming elaiosomes on ant colony performance have found inconsistent results: increased gyne (virgin queen) production [29] driven by augmented larval nutrition [30]; increased production of worker pupae but a reduction in gynes [31]; increased larval number and weight [32]; or increased investment in gyne pupae, but a reduction in egg production and male pupal weight [33]. Because these studies differed in focal ant species, plant species, and colony demographics, it is unclear how much of this variation in ant performance resulted from differences among plant species. The fact that the amino acid content of elaiosomes has been shown to influence ant preferences [34] suggests that ants could make dispersal decisions based on nutritive quality, but to date, no study has fed elaiosomes of multiple plant species to ants and compared their effects, nor examined whether ants can make decisions to collect diaspores based on their benefits.

In fact, the proximate motivation to pick up and move diaspores may have little to do with nutritional rewards. Finding that ants were not equally interested in all elaiosome components, Brew et al. [15] proposed that ants do not remove diaspores because they perceive them as food; rather, ants responded to the polar lipid fraction of elaiosomes, especially the oleyl-containing compounds oleic acid, 1,2-diolein, and triolein. Though the oleyl diglyceride 1,2-diolein is often tested for ant response (e.g., [35]), free oleic acid may be equally if not more important [15], [36]. Pfeiffer et al. [37] found that free oleic acid was the only lipid component that differed among diaspores removed at high and low rates. Ants removed diaspores of “cheater” species that contained oleic acid in the seed coat but had no removable food reward [37], suggesting that response to oleic acid can override perception of actual reward quality. Oleic acid is the most common fatty acid in coleopterans, lepidopterans, orthopterans, dipterans, hymenopterans, and blattarians [38], so it may have evolved in elaiosomes as an insect mimic [35]. Because oleic acid alone can induce foraging ants to pick up and carry objects to the nest [39], it could function as a sensory trap in elaiosomes. However, it is still unclear what roles nutrient rewards and oleic acid signaling play in motivating ants to participate in myrmecochory. Though Pfeiffer and colleagues [37] showed that reward-less plants might obtain dispersal services through oleic acid signaling, no study has examined whether diaspores that do have elaiosomes could use a similar strategy to conceal poor-quality food rewards.

We aimed to explore whether ants accurately assess partner quality, or whether plants can elicit dispersal by using false signaling or sensory traps. We examined the roles of signals and rewards in motivating dispersal of four plant species by the co-occurring ant *Aphaenogaster rudis,* which disperses the majority of seeds in this system [40]. Briefly, we tested the willingness of ants to remove diaspores of different species and examined diaspore parameters to determine which might predict ant decisions. Additionally, we provisioned lab-maintained colonies with diaspores of the same four co-occurring understory plant species and measured several components of ant colony growth. We used these data to ask: (1) Do ants decide to remove different species at different rates? (2) Are these decisions motivated by ant assessments of the rewards present in elaiosomes? (3) Or are preferences instead affected by chemical signals?

## Materials and Methods

### Study System

Our focal ant was *Aphaenogaster* cf. *rudis* Enzmann (Formicidae: Myrmicinae), a common woodland ant, and a frequent disperser of seeds in Eastern North American forests [40]. Between May and August 2010, we collected 48 queenright colonies from the Koffler Scientific Reserve at Jokers Hill (44.033°N, 79.536°W, elevation ∼300 m). This field site is the property of the University of Toronto, and our study was approved by the station director, Dr. Arthur Weis. We transferred the colonies we collected to 20×10×10 cm plastic containers. We provided the colonies with test tubes containing water and cotton, wrapped in tinfoil for darkness, and fed them two to three times a week with ∼0.5 g of standardized ant diet, modified from the Bhatkar-Whitcomb diet [41]. For the summer, we maintained the colonies indoors under ambient light and temperature conditions. In September 2010, we relocated the colonies to an environmental chamber at the University of Toronto, where they were kept during the experiment under a 14L:10D light cycle, 60% humidity, and 25°C daytime and 15°C night-time temperatures, simulating July conditions at the site of collection.

We collected diaspores of *Sanguinaria canadensis* L. (Papaveraceae), *Asarum canadense* L. (Aristolochiaceae), *Trillium grandiflorum* (Michx.) Salisb., and *T. erectum* L. (Melanthiaceae) from the same general locations as we collected ants. None of the species used in this study are endangered or protected. All four of these species have a single flower and produce a single fruit containing many seeds with conspicuous elaiosomes. We collected fruits and seeds when the fruits began to open or abscise naturally. This occurred asynchronously (*S. canadensis*, mid-June; *T. grandiflorum,* mid-July; *T. erectum*, mid-August), and lasted approximately two weeks for each species. Fruits of *A. canadense* never appeared to open, but were collected in mid- to late-June as many *A. canadense* fruits started to disappear, presumably removed by frugivores. Seeds were stored at −20°C until use.

### Removal Trials

Twenty-five colonies were initiated into the experiment on 5-Oct-2010 and 23 more on 12-Oct-2010. We first transferred 50 haphazardly selected workers and the queen into new containers (cylindrical, ∼10 cm in radius, 10 cm in height). Some colonies had matured gynes that had lost their wings; these could not be reliably distinguished from the founding queen and thus were included in the transfer. We discounted one worker for each additional gyne. We initially provided the colonies with two nesting tubes; these were replaced as they dried or became moldy. Three times a week, we fed each colony 0.2±0.02 g of standardized diet and removed food remaining from the previous feeding.

Because ants feed elaiosomes to larvae [19], we waited until eggs had hatched before initiating our trials. On the Tuesday following the first observation of larvae, each colony participated in seed removal trials. We presented every colony with one diaspore of each species, in random order. We placed the diaspore in the center of the colony’s container on a 4-cm circle of aluminum foil (the “depot”). We recorded how many ants were foraging when the diaspore was presented; how long it took the ants to discover the diaspore; how many times they antennated the diaspore before removing it; and how long they took to remove it from the depot. We measured the time from discovery to removal with a five-minute cut-off; any diaspores not removed within five minutes of discovery were discarded. When an ant did remove a diaspore, we temporarily removed that worker and permanently removed that diaspore from the colony. We then continued trials with the remaining diaspore species. All four diaspores were presented within 45 minutes of each other, but the removal of workers that carried diaspores ensured that most workers encountering new diaspores were naïve, and order of presentation did not affect removal (results not shown). Each diaspore was presented on a new depot.

Here, we opted for sequential presentation of individual diaspores; with asynchronous fruiting times, it is extremely unlikely that a foraging worker in a natural setting would ever be faced with the choice to disperse a seed of one species over another. Instead, what is relevant for assessing the quality of service ants provide to plants is whether workers decide to collect seeds they encounter and how long they take to do so. Thus, we are following similar studies that have used no-choice paradigms to examine whether workers decide whether or not to collect single food items based on their nutritive quality (e.g., [42], [43]).

### Diaspore Analysis

To examine whether seed or elaiosome size affected whether ants pick up and move the diaspore, we weighed each diaspore used in the removal trials. We took the wet mass of each diaspore, then separated the elaiosome from the seed and measured the wet mass of both separately.

To determine the average free oleic acid content of the elaiosomes, we haphazardly chose 12 diaspores per species from a separate pool, and removed their elaiosomes for analysis by combined gas chromatography-mass spectrometry (GC-MS). Each elaiosome was homogenized in methanol. An aliquot of the homogenate was transferred to a test tube containing the internal standard and ultra pure water was added. The oleic acid was extracted with iso-octane, and the iso-octane phase was transferred to another test tube before re-extracting the aqueous phase with iso-octane. The combined iso-octane extracts were evaporated under nitrogen gas. The dried residue was derivatized with diisopropylethylamine and pentafluorobenzyl bromide at room temperature. After 20 minutes, the sample was taken to dryness under nitrogen gas and the residue reconstituted in iso-octane. A volume of 1 µL was injected onto the GC-MS system. A nine-point standard curve was processed in the same way as the seed samples. Separation was conducted by GC (Agilent 7890A) on a fused silica SP-2380 Supelco column (30 m×0.25 mm internal diameter, 0.2 µm film thickness) with helium as the carrier gas at a flow rate of 1 mL min^−1^. An initial temperature of 150°C was held for 1 min, followed by ramping to 270°C at 12°C min^−1^ then to 275°C at 40°C min^−1^, where the temperature was held for 3 min. The GC was coupled to a quadrupole mass spectrometer (Agilent 5975C), and the oleic acid peak was identified, with heptadecanoic acid as an internal standard.

### Ant Colony Performance

To determine whether ants’ rate of diaspore removal might be explained by the nutritive quality of the elaiosomes, we provided colonies with diaspores as a food source. Following removal trials, we assigned colonies at random to one of the following treatments: control (n = 9), *A. canadense* (n = 10), *S. canadensis* (n = 10), *T. grandiflorum* (n = 10), or *T. erectum* (n = 9). On the day following the diaspore choice trials, we gave the colonies 0.16±0.005 g of standardized diet, plus enough diaspores of the corresponding plant species to make up the equivalent of 0.04 g (wet wt.) of elaiosomes. Control colonies were fed the usual 0.2±0.02 g of standardized diet. We repeated this treatment once more, two weeks later. Otherwise, colonies in all treatments were fed 0.2±0.02 g of standardized diet three times a week for the duration of the experiment. We thus provided colonies with an amount of diaspores consistent with a single ant colony being able to monopolize one or two fruits. A similar quantity of elaiosomes has elicited an effect in other work [29], [30].

To track worker mortality throughout the experiment, we counted and removed dead workers weekly. Occasionally, corpses were dismembered or consumed by other workers, so we probably slightly underestimated mortality. Ten weeks after a colony was initiated into the experiment, we measured the number and mass of brood in the colony. We removed workers, brood, and the queen from the nest tubes, and weighed eggs and larvae on a microbalance, then counted them under a dissecting microscope.

### Statistical Analysis

We used generalized linear mixed models to test for effects of diaspore species and number of active workers on time to diaspore discovery (log-transformed, Gaussian errors) and number of antennations (negative binomial errors), with colony identity as a random effect. The diaspore species by number of active workers interaction was not significant in either case and was excluded [44]. Multiple comparisons were Tukey-adjusted.

To account for censored data, we analyzed the time between discovery and removal using survival analysis. We initially used univariate analyses on a number of potential explanatory variables, before including in the final model only those predictors that yielded a significance of p<0.25. In univariate analyses, we tested colony identity (each colony was tested with one diaspore of each species), diaspore species, order of presentation, number of active foraging workers, diaspore mass, seed mass, elaiosome mass, and the ratio of elaiosome to seed mass. Only diaspore species, colony (as a blocking effect), and number of active workers yielded a significance of p<0.25 and were included in the final model. The assumption of proportionality was confirmed for number of active workers and diaspore species by re-running the model using time-dependent covariates. We used Tukey-adjusted p-values for multiple comparisons. We compared elaiosome mass, seed mass, and the ratio of the two among species using generalized linear models (GLMs). Then, to confirm whether any of the size metrics influenced removal time, we also ran survival analysis models including diaspore species, colony, and number of active workers, along with elaiosome mass, seed mass, and elaiosome to seed mass ratio individually. Oleic acid content could not be included, as we measured oleic acid concentrations in a different subset of diaspores.

All measures of colony performance taken 10 weeks after treatment (number of eggs, larvae, and pupae; log-transformed total mass of brood; number of worker deaths since treatment) were analyzed with GLMs, with treatment as the main effect and colony size at the start of treatment as a covariate controlling for underlying variation in ant colony condition. The treatment by covariate interaction was never significant, and was thus excluded. The brood mass model used Gaussian errors; all other variables were tested in models with negative binomial errors.

GC-MS results (oleic acid content per elaiosome and oleic acid concentration) were log-transformed and analyzed in GLMs with Gaussian errors. Because we measured the oleic acid content of different elaiosomes from those used in removal trials, it was not possible to directly regress removal on oleic acid concentration, as we did with size measures.

Survival analyses were conducted in SAS (SAS Corporation, v. 9.2); all other analyses used R (v. 2.15.2).

## Results

### Removal Trials

**Figure 1:**
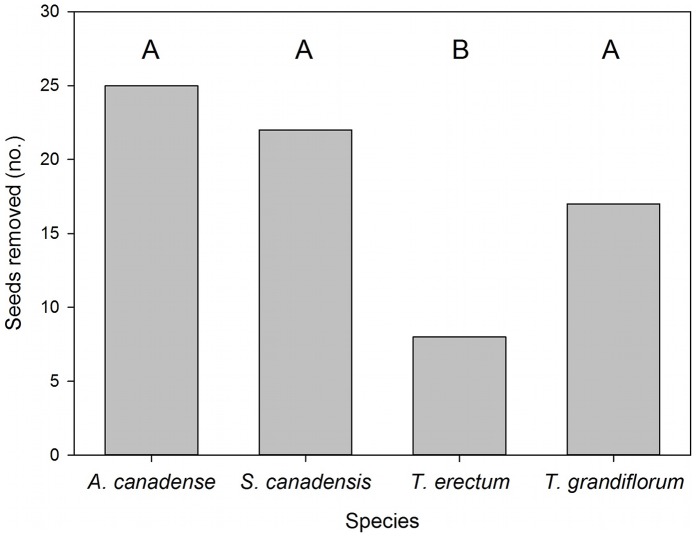
Seed removal by *Aphaenogaster rudis* workers. Numbers of diaspores of *Asarum canadense, Sanguinaria canadensis, Trillium erectum,* and *Trillium grandiflorum* removed by workers within one minute, out of a total of 48 presented to colonies. Time to removal was measured from the time workers first discovered a diaspore. Different letters indicate statistically significant differences in removal, as assessed by full survival analysis models (see text).

Ants removed some diaspore species faster than others, even though they discovered diaspores at the same rate. After workers discovered diaspores, plant species identity affected time to removal (Fig. 1; Wald X^2^ = 33.79, df = 3, p<0.0001; hazard ratios: *A. canadense = *2.29, *S. canadensis = *1.56, *T. grandiflorum* = 1.00, *T. erectum = *0.34). Ants removed *T. erectum* diaspores significantly more slowly than they removed those of the other species we tested (Fig. 1; Tukey-adjusted comparisons with: *A. canadense*, p<0.0001; *S. canadensis*, p<0.0001; *T. grandiflorum*, p = 0.043). Ants also removed *T. grandiflorum* diaspores marginally slower than those of *A. canadense* (p = 0.053). Greater numbers of active workers were associated with faster removal (Wald X^2^ = 4.98, df = 1, p = 0.026) and colony identity (Wald X^2^ = 68.59, df = 47, p = 0.022) also influenced removal. Diaspore species also affected the amount that ants antennated diaspores before removing them (mean ±1 SE was 3.44±0.45 for *A. canadense*, 4.33±0.43 for *S. canadensis*, 5.15±0.48 for *T. erectum*, and 4.33±0.49 for *T. grandiflorum*; X^2^ = 13.96, df = 3, p = 0.003). Specifically, workers antennated *T. erectum* diaspores significantly more often than *A. canadense* diaspores (Tukey-adjusted p = 0.010). Number of active workers did not affect antennations (X^2^ = 1.54, df = 1, p = 0.215). Ants discovered diaspores faster when more workers were active (X^2^ = 14.85, df = 1, p = 0.0001), but the time it took ants to discover a diaspore did not vary among diaspore species (mean ±1 SE was 59.9 s ±18.2 s for *A. canadense*, 59.9 s ±15.0 s for *S. canadensis*, 58.0 s ±12.2 s for *T. erectum*, and 42.7 s ±7.9 s for *T. grandiflorum*; X^2^ = 0.60, df = 3, p = 0.897).

### Diaspore Analysis

**Figure 2:**
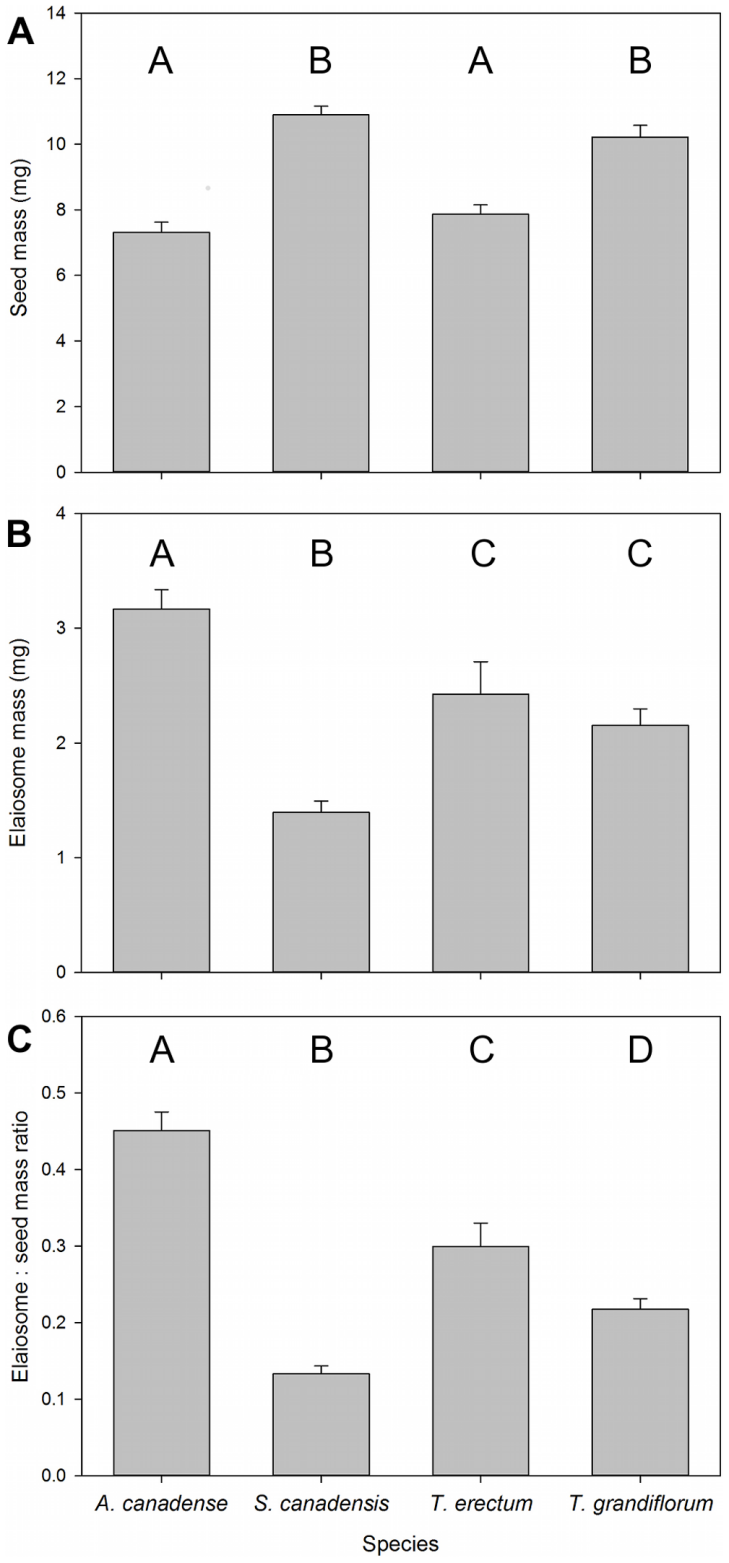
Sizes of diaspores presented to ants. Mean (± SE) a) seed mass, b) elaiosome mass, and c) ratio of elaiosome to seed mass for seeds collected by ants in removal trials. Different letters indicate statistically significant differences (Tukey-adjusted p≤0.05).

Ants did not collect diaspores based on elaiosome or seed mass. Diaspores did differ in seed mass (Fig. 2a; X^2^ = 95.34, df = 3, p<0.0001), elaiosome mass (Fig. 2b; X^2^ = 46.07, df = 3, p<0.0001), and the ratio of the two (Fig. 2c; X^2^ = 122.96, df = 3, p<0.0001). Diaspores of *A. canadense* had significantly larger elaiosomes than the two *Trillium* species, which in turn had elaiosomes significantly larger than those of *S. canadensis* (Fig. 2b). The seeds of *A. canadense* and *T. erectum* were also significantly smaller than those of *T. grandiflorum* and *S. canadensis* (Fig. 2a), and so the ratio of elaiosome to seed mass was highest for *A. canadense*, followed by *T. erectum, T. grandiflorum*, and then *S. canadensis* (Fig. 2c). However, these differences did not predict removal rates. When individually included in survival analysis models analyzing the effect of diaspore species on removal by ants, neither seed mass (Wald X^2^ = 0.016, df = 1, p = 0.898), elaiosome mass (Wald X^2^ = 0.63, df = 1, p = 0.427), nor the ratio between the two (Wald X^2^ = 1.39, df = 1, p = 0.239) significantly predicted time to removal. Though diaspores did vary in size, mass could not explain ants’ removal of different species.

**Figure 3:**
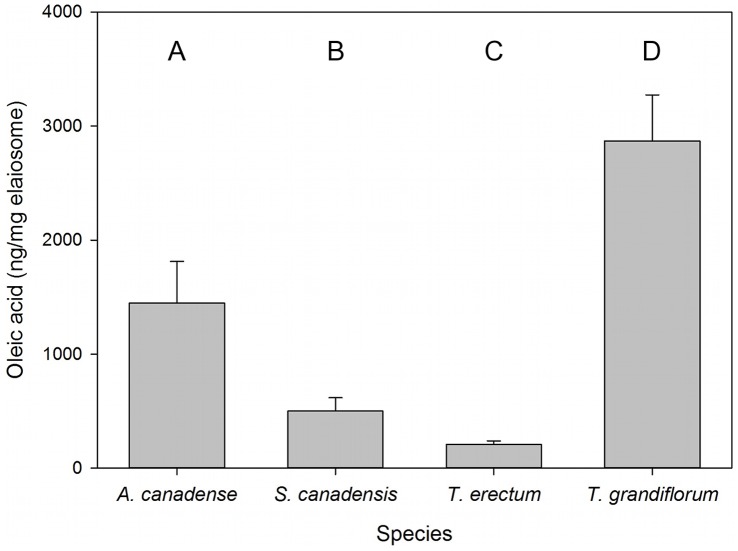
Oleic acid content of the focal myrmecochorous species. Mean (± SE) concentration of free oleic acid in elaiosomes. Different letters indicate statistically significant differences (Tukey-adjusted p≤0.05).

Species also varied in both the amount of oleic acid (free fatty acid fraction) per elaiosome (X^2^ = 482.81, df = 3, p<0.0001), and the concentration of oleic acid per mass of elaiosome tissue (Fig. 3; X^2^ = 93.3, df = 3, p<0.0001). Eight of 12 *T. erectum* elaiosomes had oleic acid concentrations that fell below the 20 µg minimum calibration threshold and thus exact values are not quantitatively accurate; however, since they fell below this minimum value, the qualitative interpretation of these values is sound.

### Ant Colony Performance

**Figure 4:**
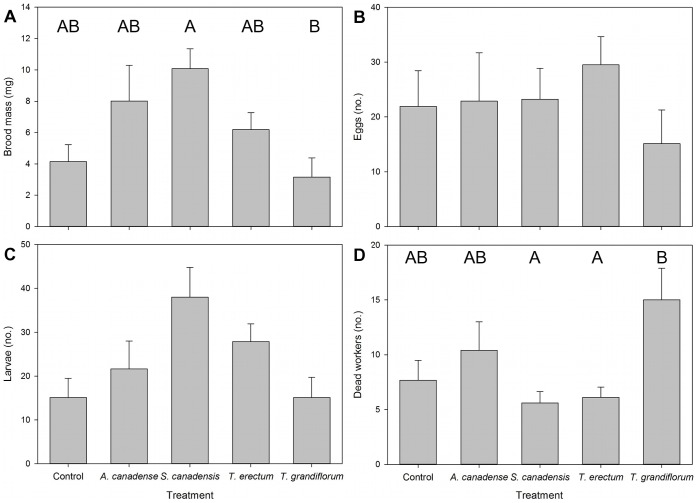
Effects of diet treatment on colony performance. Mean (± SE) a) brood mass and b) numbers of eggs, c) larvae, and d) dead workers 10 weeks after treatment. Different letters indicate statistically significant differences (Tukey-adjusted p≤0.05).

Diaspore treatment significantly affected several metrics of colony performance. Brood mass varied significantly among treatments (Fig. 4a; X^2^ = 15.8, df = 4, p = 0.003). Colonies that were larger at the start of treatments (i.e. those that had fewer of the initial 50 workers die) also had greater masses of brood (X^2^ = 8.85, df = 1, p = 0.003). Colonies provided with *S. canadensis* diaspores had a significantly greater brood mass than those provided with *T. grandiflorum* (Tukey-adjusted p = 0.002) and marginally significantly more than those in the control treatment (Tukey-adjusted p = 0.093). Colonies in the *A. canadense* treatment also had marginally greater brood masses than those in the *S. canadensis* treatment (Tukey-adjusted p = 0.098). Treatment did not significantly affect numbers of eggs (Fig. 4b; treatment: X^2^ = 2.35, df = 4, p = 0.673; colony size: X^2^ = 2.43, df = 1, p = 0.119) or larvae (Fig. 4c; treatment: X^2^ = 7.14, df = 4, p = 0.129; colony size: X^2^ = 12.93, df = 1, p<0.001). Only one colony, in the *S. canadensis* treatment, matured a larva into a pupa. Worker mortality was also significantly affected by treatment (Fig. 4d; X^2^ = 15.93, df = 4, p = 0.003): more workers died in the *T. grandiflorum* treatment than in the *S. canadensis* (Tukey-adjusted p = 0.006), *T. erectum* (p = 0.044), and control (p = 0.067) treatments, although the p-value of the latter comparison is marginal.

## Discussion

Though a handful of studies have demonstrated that ant colonies benefit from dispersing seeds [29]–[33], ours is the first to demonstrate that not all diaspores provide equal rewards: colonies performed significantly worse when fed *T. grandiflorum* diaspores, compared to *S. canadensis*. Furthermore, our results show that although ants differ in their dispersal of different diaspore species, their choices are not based solely on an assessment of the rewards in elaiosomes; extreme levels of the signaling molecule oleic acid may overwhelm ant perceptions of quality.

Foraging ants removed about half of the diaspores of *A. canadense* and *S. canadensis* within one minute of making contact with them (Fig. 1). Ants removed *T. grandiflorum* diaspores somewhat more slowly, although not significantly so. However, ants removed *T. erectum* diaspores significantly more slowly: approximately two-thirds were not removed within the five-minute observation period. Additionally, even when ants did remove *T. erectum* diaspores, it was only after a greater number of inspections, indicating a lower probability of removal for each visit. These differences arose in a lab setting in which seeds were placed close to nests and ants were abundant. In nature, with lower densities of ants, these differences could be further exaggerated and thus potentially affect seed fates.

Discrepancies in the removal of diaspores did not arise from differences in detection or attraction from a distance; detection time did not differ among plant species. This suggests that distance-acting cues, be they visual or olfactory, did not influence ant choices. Past research also failed to find an effect of color on diaspore choice by *Myrmica ruginodis* [27], and Sheridan et al.’s [45] physiological and behavioral examination of the perception of *A. canadense* diaspores by four ant species, including *A. rudi*s, also found that the ants responded to gustatory, not volatile cues. Gas chromatography did not detect any volatile chemicals being emitted from the diaspore species we studied here (K. Turner, unpubl. data). The advantages of remaining inconspicuous to seed predators may explain why myrmecochorous plants have not evolved to attract foraging ants with volatiles. Instead, plants apparently rely on chance encounters with abundant and active foraging ants. In our trials, ants appeared unaware of diaspores until they contacted them directly.

Removal differences arose after ants gathered information through contact. However, ants did not appear to make foraging decisions based on diaspore size; though diaspores varied in size, we were unable to detect any influence of diaspore mass, elaiosome mass, or the ratio of the two on removal rates. This conflicts with past findings of differences in rate of removal based on elaiosome size [17] or elaiosome:seed load ratio [16]. The foraging decicions of *A. rudis* may only be responsive to a wider range of seed or elaiosome sizes. Other studies have also found that size does not completely predict removal [46], [47].

Instead, in some cases, ants may have responded to the quality of elaiosome rewards. Provisioning ant colonies with equal masses of different species of elaiosomes did have disparate consequences for colony growth. Consuming *A. canadense* did not yield any significant increases in colony performance compared with the control, but also yielded no apparent cost in worker mortality or brood production. The diaspores of this species thus appear to have been at least worth the cost of foraging on them in lab conditions. Colonies fed *S. canadensis* diaspores did even better: they maintained a low worker death rate, and had marginally larger brood clutches than colonies maintained only on the control diet. Their larvae were probably also heavier, since treatment differences arose in total brood mass rather than in larval numbers. The larvae may have been developing more quickly, or they may have simply been larger and therefore more likely to develop into gynes [48]. In a field study, Bono and Heithaus [30] similarly found that *A. rudis* larvae that fed on more *S. canadensis* elaiosomes were more likely to mature into gynes, and Morales and Heithaus [29] likewise showed *S. canadensis* to be beneficial to field colonies of *A. rudis.* Our experiment thus yielded similar results in a lab context, and showed that workers were able to identify *S. canadensis,* as well as *A. canadense,* as adequate food sources, and provide rapid dispersal.

In contrast, ant removal of *T. grandiflorum* and *T. erectum* diaspores is not easily explained by the nutritive rewards in their elaiosomes. Almost twice as many workers died in colonies that received *T. grandiflorum* diaspores as in colonies that received *S. canadensis, T. erectum,* or no diaspores. Brood mass was also significantly less in the *T. grandiflorum* than in the *S. canadensis* treatment. *Trillium grandiflorum* elaiosomes may be such poor-quality food that they failed to make up the energetic costs of foraging for them, or they may produce some component that is toxic to ants (possibly as a defense against microbes or other antagonists, as in toxic floral nectar; [49]). Yet *T. grandiflorum* diaspores were removed as rapidly as *S. canadensis* diaspores, a discrepancy that may be explained by signaling: *T. grandiflorum* had concentrations of oleic acid several times greater than those of the other three plant species. Conversely, *T. erectum* had the lowest oleic acid concentration, and ants were unlikely to remove *T. erectum* diaspores even though they were similar to *A. canadense* diaspores in food rewards. In making foraging decisions, *A. rudis* ants may use oleic acid content as a proxy for food quality, which is likely beneficial in most cases, given the abundance of this lipid in ant food sources [38]. Indeed, foraging ants in this habitat would often encounter species like *S. canadensis* that use oleic acid signaling and are rewarding. The false signaling of *T. grandiflorum* may thus be enhanced by the fact that its seeds are released shortly after those of *S. canadensis* and other more beneficial species. Since oleic acid triggers a carrying response in foraging ants [39], its presence may function as a sensory trap [11], which may have allowed *T. grandiflorum* to still be dispersed by workers in spite of a reward which provided a low benefit to them. Pfeiffer et al. [37] showed that diaspores with no reward elicited some dispersal using oleic acid signaling; here, we have directly examined reward quality of myrmecochorous species, and shown that oleic acid may misinform ants even among plants that do provide a removable reward. Other signaling and contextual factors are likely important, but given substantial literature support [15], [35]–[37], [47], [50], [51], the extremely high and low levels of oleic acid found in *T. grandiflorum* and *T. erectum*, respectively, probably interfered with ants’ assessments of the actual quality of food rewards. Though ants do exert selection on reward quantity [52], oleic acid signaling appears to modify workers’ decisions.

If our findings in a lab setting are reflective of the plant rewards and ant preferences in nature, the evolution of signals in *Trillium* is at first confusing: it is clear how deceptive signaling could be advantageous for *T. grandiflorum*, but why would *T. erectum* poorly signal its relatively high quality? Schaefer and colleagues [10] emphasized that plants (unlike animals) cannot use behavior to switch signals in the presence of more mutualistic or more antagonistic partners, so perhaps *T. erectum* hides its nutritive quality from seed predators, at the expense of also hiding from seed dispersers such as *A. rudis*. However, *T. erectum* does make its quality apparent to some seed dispersers – *Myrmica punctiventris* ants readily collect their diaspores [46]. *Trillium erectum* may thus be using fine-tuned “private channels” to target quality signals to specific receivers; Gammans et al. [27] similarly showed that *Ulex* spp. selectively attract seed dispersers but not seed predators. Here, *T. erectum* may repel *A. rudis* in favor of other, superior dispersers, which may include co-occurring *Myrmica* spp. or other ant species; ‘myrmecochorous’ seeds may even receive higher quality dispersal from harvestmen [46], gastropods [53], or vespid wasps [54].

Several other questions about the evolutionary dynamics of these partnerships remain unanswered. On the plant side, understanding the relative costs of producing oleic acid and ant-rewarding nutrients could shed light on adaptive signal evolution in myrmecochorous plants. Specifically, even if *T. grandiflorum* elicits dispersal through deception in the field, as it does in a lab setting, we need to know whether making signals instead of rewards is a cost-saving strategy before we label *T. grandiflorum* a “cheater.” On the ant side, if oleic acid misinforms *A. rudis* workers, why do these ants still preferentially disperse oleic acid-rich diaspores? Even though we have shown that under controlled conditions *T. grandiflorum* can negatively affect colony growth, in the context of natural colony demographics and dietary backgrounds, misinformed diaspore choices may have negligible effects on colony fitness. Furthermore, oleic acid is very common in ant foods [38], and thus reducing response to oleic acid may be counter-adaptive overall.

Conducting our experiment in the lab allowed us to control background diet and colony structure, and to eliminate the effects of predation and spatial arrangement, which thus allowed us to pinpoint differences in seed removal and colony performance driven by interspecific differences in elaiosomes. Further, it allowed us to precisely examine effects on worker mortality and brood production, which would be impossible in field settings without disruptively sampling colonies. These considerations are likely why colony performance is commonly measured in the lab in studies of myrmecochory [31]–[33], other ant-plant interactions [55], [56], and ant dietary ecology [42], [43].

However, the dietary needs of our *A. rudis* colonies would likely differ in a field setting, and even in the field would vary with colony size, the presence of larvae and sexuals, abiotic conditions, and the availability of other food sources. Though our experimental conditions closely replicated temperature and light conditions at the site of collection, these other factors could affect ant responses to food sources. It has been repeatedly demonstrated that ants can adjust their foraging decisions to reflect changed dietary and demographic contexts, collecting the food most beneficial to them in that context [42], [43], [56], [57]. This ability, however, depends on ants’ ability to accurately assess the contents of food sources. Here we have shown that foraging ants were in fact unable to make such an assessment of the nutritive quality of elaiosomes. Given the ants’ dietary and demographic context, collecting *S. canadensis* diaspores led to an increase in colony growth and fitness, diaspores of *T. grandiflorum* had negative impacts on those same metrics, and two other myrmecochorous species had intermediate impacts. Yet foraging ants were unable to recognize the contents of some food rewards as meeting or not meeting their current dietary needs. Instead, oleic acid signaling appears to have interfered with worker ants’ decisions, disproportionately attracting ant interest in some cases (*Trillium grandiflorum*), and repelling them in others (*T. erectum*). Recent studies have demonstrated that the rewards of ant-dispersed seeds may be adapted to nourish ants [28], [29], [31]–[33], and that co-evolution may have constrained the amount of reward provided by diaspores [52]. However, our results caution that the nutritive rewards elaiosomes provide may not predict ant responses.

This study extends the sparse literature on the rewards that ants accrue through participation in myrmecochory [29], [31]–[33], and indeed the very sparse literature on the benefits ants gain from ant-plant partnerships in general [55]. Furthermore, it is the first to demonstrate that different myrmecochorous plant species provide different levels of reward. However, it also shows that these differences in apparent partner quality do not completely predict whether ants will remove seeds. Instead, workers of *Aphaenogaster rudis*, a major seed disperser, are unable to accurately assess the quality of elaiosome rewards and disperse seeds accordingly. Signaling apparently influences their decisions, and our results support the hypothesis that oleic acid is important in this role. Thus, ants’ seed dispersal behaviors are simultaneously influenced by both rewards and signals produced by their partners.
